# Supplementary material for: Global prevalence, mortality, and main characteristics of HIV-associated pneumocystosis: A systematic review and meta-analysis
Source: PLoS One. 2024 Mar 25;19(3):e0297619. doi: 10.1371/journal.pone.0297619 (PMC10962827; doi:10.1371/journal.pone.0297619)
Supplement: S1 Fig — (DOCX) [file pone.0297619.s002.docx]

**Supplementary Figure 1.** L’Abbe and Funnel plots for prevalence OR of HAP patients.
